# Endodontic Tendencies in a Very-Low-Income Population Area of Northeastern Brazil

**DOI:** 10.1155/2019/9543593

**Published:** 2019-09-05

**Authors:** Maria Tereza Pedrosa Albuquerque, Joice Velames Silva, David Junio Oliveira Poppe, Crislaine Santana Santos, Tainá Pires Santos, Eliseu Aldrigh Münchow, Juliana Yuri Nagata

**Affiliations:** ^1^Department of Clinical Dentistry, Endodontics, Federal University of Bahia, Av. Araújo Pinho, 62, Salvador, BA 40110-040, Brazil; ^2^Dentistry Department, Endodontics, Federal University of Sergipe, Av. Gov. Marcelo Déda, 300-São José, Lagarto, SE 49400-000, Brazil; ^3^Federal University of Rio Grande Do Sul, Department of Conservative Dentistry, Rua Ramiro Barcelos, 2492, 90035-004 Porto Alegre, RS, Brazil

## Abstract

**Aim:**

To gather information regarding endodontic treatment protocols and continuing education attendance of dentists in some cities located in Northeastern Brazil (Sergipe and Bahia States), a region that comprises a very-low-income population.

**Methods:**

A questionnaire containing different questions (e.g., routine treatment protocols, the use of new technologies, time required to conclude the treatment, and attendance in an endodontic continuing education) was distributed to 250 dentists. The collected data were analyzed through descriptive statistics and Poisson regression (*p* < 0.05).

**Results:**

A total of 199 dentists practicing endodontic treatment in the cities of Aracaju (*n* = 58), Salvador (*n* = 83), and towns of Estância (*n* = 8), Itabaiana (*n* = 16), Itabaianinha (*n* = 5), Lagarto (*n* = 10), Ribeirópolis (*n* = 3), Simão Dias (*n* = 6), and Tobias Barreto (*n* = 10) participated in this research. Most of the respondents have concluded their graduation in Dentistry in less than 10 years (62.3%), with nearly 75.4% of the sample having attended postgraduation education in the field. Regarding treatment protocols, the frequency for using rotary/reciprocating systems and for always using rubber dam isolation during root canal treatment (RCT) was 78% and 62%, respectively, which was also more prevalent among dentists who attended a continuous education program (*p* < 0.05). Lastly, the professionals who attended (*p* < 0.05) to a continuous education program in Endodontics were more frequently associated to performing incisors and molars RCT in one clinical appointment (*p* < 0.05).

**Conclusions:**

The results of this survey indicated that even in an area where most of the population has low-income conditions, the professionals are seeking for knowledge by attending to postgraduation programs, following the new tendencies in Endodontics with most of the professionals having employed technological resources.

## 1. Introduction

Endodontic treatment has achieved increasing success rates (86–90%) in the last decades [1–3]. This improvement has been mainly attributed to the advances in emerging techniques/materials and also to the interest of the professionals on acquiring knowledge (e.g., *lato sensu* and *stricto sensu* programs and the attendance to dental conferences) [2, 3]. The development of new instruments (e.g., nickel-titanium—NiTi files), devices (e.g., electronic foraminal locators, digital radiograph, termoplastified obturation, clinical microscopy, and passive ultrasonic irrigation), and reparative materials (e.g., biomaterials and tricalcium silicate sealers) has contributed to make the endodontic treatment more predictable, efficient, and accurate [4–6]. Moreover, technology has also provided a more comfortable experience to the patient, saving chair-time and reducing the operational costs to the dentist [3, 4].

With the advent of these technological innovations, professionals have progressively detected the need for searching academic/clinical qualification in the Endodontic field, increasing the number of postgraduate dentists. Accordingly, the Endodontic field in Brazil encompasses nearly 15.622 professionals, representing the second biggest specialty in the country [7]. However, there is still uncertainty whether in regions of low-income population such as the Northeastern area of Brazil, the professionals are following the endodontic technologies that have been emerging. Also, from the best of our knowledge, there is no previous report in the literature concerning the seek for knowledge by professionals performing root canal treatment (RCT).

Sergipe is located in the Northeastern region and represents the smallest state of Brazil, covering only 98 endodontists (1%) registered in the National Federal Board [8]. The low number of endodontists in Sergipe might be related to the fact that there is only one registered continuing education program in Endodontics (CEPE) in the entire state, from a total of 31 programs widespread in the country [8]. On the other hand, Bahia represents one of the biggest states in Brazil containing approximately 8 CEPEs [8]. Despite the number of CEPEs in Brazil, a master program has been related to high costs and longer duration, which are both unfavorable conditions for many professionals. However, the upward access to technology, especially the NiTi rotary/reciprocating files and apical locator, is heading the dentists to seek for know-how in Endodontic practices by means of short-duration and low-cost programs. In this scenario, a growing number of short-duration and low-cost programs in the field of Endodontics have emerged, which provide theoretical and clinical practice that deal with less complex clinical cases.

Beyond this scientific and technological progress, there are also biological controversies in the literature and technical preferences by each professional that may create doubts concerning the most appropriated protocol to be followed by the professionals that perform RCT. Furthermore, no study so far has investigated the professional qualification status and how far the changes in endodontic technique have been incorporated into daily practice in two states (Sergipe and Bahia) in the Northeastern Brazil. Hence, this study aimed to gather information about routine endodontic treatment protocols and their relation to professional qualification by dentists in some cities in Northeastern Brazil, a region that comprises a very-low-income population.

## 2. Materials and Methods

This cross-sectional study was previously approved by the Research Ethics Committee of Federal University of Sergipe (acceptance number: 51153615.9.000.5546).

In view of the widespread technologies and materials available to optimize the endodontic treatment, a questionnaire was designed to evaluate routine preferences (traditional and/or advanced) in the root canal therapy of dentists in some cities of the Northeastern area of Brazil, including Sergipe and Bahia states. The assignment was divided into two parts: first one was composed by questions concerning demographic and professional qualification and the second part was related to the protocols of endodontic treatment.

A total of 250 questionnaires were randomly distributed among dentists that perform endodontic treatment in the states of Sergipe and Bahia, encompassing the cities of Aracaju, Lagarto, Itabaiana, Estância, Itabaianinha, Tobias Barreto, Simão Dias, Ribeirópolis, and Salvador. According to the Federal Dentistry Council of Brazil, the total number of dentists working in the cities of Sergipe and Bahia totalizes 1.775 and 12.395, respectively [9]. In the present research, all the questionnaires were delivered personally to the dentists that reported to perform endodontic treatment in the period of 2017 and 2018, representing 8.4% of the foregoing professionals.

All the participants were invited to answer the questionnaire. After their acceptance, the respondents received a briefly explanation regarding the survey, emphasizing its confidentiality (no identification). The professionals also had the option to opt out of answering the questionnaire. The questionnaires were collected on the next day, immediately separated from the consent form, and put in an envelope to keep anonymous the identity of the participants. Descriptive statistics and Poisson regression test in a significance level set as *P* < 0.05 were used to analyze the collected data.

## 3. Results

From a total of 250 questionnaires, only 199 dentists full filled it, representing a 79.6% of full-response rate.  Table 1 shows that the most representative respondents were female dentists (59.8%), with the most prevalent age ranging from 20 to 30 years old for both genders (42.2%). A greater number of professionals attended public dental schools during their graduation (57.8%). Also, most of the respondents have concluded their graduation in dentistry in less than 10 years (62.3%), with nearly 18% of the sample displaying more than 20 years of experience in the field. Concerning continuous education in Endodontics, most of the interviewed samples (75.4%) attended a postgraduate program, which was distributed within shorter programs with a smaller duration (less than 1 year) (28.7%) and longer programs of Master Science in Endodontics (47%).

In the second part of the questionnaire, it was investigated endodontic treatment protocols performed by the professionals (Table 2). It was observed that most of the dentists (75.9%) always use rubber dam isolation during the root canal treatment (RTC). Approximately half of the whole sample (58.3%) affirmed to use NiTi endodontic files/systems during RCT, especially reciprocating files (53.3%). Only 40.2% of the dentists affirmed to have attended to a qualification program (e.g., dental conferences, immersion programs, short-term programs, and hands-on) in the past two years. Regarding technological resources, operating an apical locator to delimit the working length was reported by 62.8% of the professionals. Conversely, most of the participants mentioned to use manual stainless-steel endodontic files (86.9%) and Gates Glidden burs (67.3%) during RCT. Concerning the most prevalent technique to accomplish RCT, the crown-down technique was cited as the preferred method by most of the professionals (83.9%). During root canal shaping, most of the respondents (90.4%) reported to use sodium hypochlorite (NaOCl) as the chemical solution to rinse the root canal during the instrumentation, 10% stated to use chlorhexidine, 25.1% saline solution, and 84.9% ethylenediaminetetraacetic acid (EDTA) (Table 2). Besides the use of irrigants, root canal decontamination may also be enhanced through the use of intracanal medications, and calcium hydroxide paste was frequently used by 74.3% of the respondents, which mostly reported to maintain the intracanal medication into the root canal for 4–7 days (50.3%) (Table 2).

Table 3 shows the crude analyses of some factors (e.g., graduation dental school, years since graduation, use of rotary systems, use of rubber dam isolation, and the teeth to be treated in one appointment in different pathologic conditions) compared to a fixed variable of attending a continuous education program in Endodontics (CEPE). It was observed that the use of rotary/reciprocating systems was 78% more prevalent among dentists who attended a CEPE compared with those who have not carried out any postgraduation (*p* < 0.05). Similarly, the prevalence for always using rubber dam isolation during RCT was 62% higher among postgraduated dentists as compared with the professionals who have declared never using absolute isolation (*p* < 0.05). Lastly, attending a CEPE showed at least thrice and twice the prevalence of performing RCT of incisors and molars in one clinical appointment, respectively, when compared to dentists who do not perform such protocols (*p* < 0.05). From the dentists who perform RCT in incisors in one appointment, the majority usually takes less than 1 hour to finish the treatment; conversely, in the case of molars, most of the dentists take more than 1 hour to complete the RCT.

## 4. Discussion

Seeking knowledge is essential to improve skills in Dentistry. So that, this study investigated whether professionals have been enhancing their qualification in Endodontics within the last years; also, data regarding RCT protocols practiced by general dentists and Endodontists in the states of Sergipe and Bahia (Brazil) were collected, including preferences of materials, instruments, and techniques used during the treatment. This study used a different stand-point compared to the great majority of studies that focused on highly-populated cities [10–14]. Indeed, the Northeastern area of Brazil was chosen here since it presents extreme poverty (i.e., half of the population and their families earn less than $250 dollars per month, which hampers their affordability for health services) [15]. Hence, the current study aimed to explore the smallest state of the country (Sergipe) that holds only three universities offering Dental School qualification (two publics and one private), which graduate annually approximately 150 professionals [7]. Additionally, the biggest state of the northeastern area (Bahia), which is also the 5^th^ largest state of the country, was also included in this research. Bahia encompasses nearly 16 Dental Schools, graduating annually ∼960 professionals that actuate in all areas of Dentistry [7].

Considering the foregoing numbers and the full-response rate of 199 respondents, 47% of the professionals attended at least one CEPE, opposed by 53% professionals that were general dentists performing endodontic treatments. Comparing to other countries, in Turkey, only 1.1% of the dentists are specialists in Endodontics [16]. These results show that a greater number of dentists (47%) have been seeking for knowledge in the Endodontics field even in the northeastern region of the country, which shelters the less favored population.

Another profile feature assessed in this study was the interval between the professional's graduation and their clinical practice. It was observed that most of the professionals have graduated in the past 10 years (62.3%), showing a higher frequency of recent graduated professionals actuating in Endodontics. These data emerge in accordance with the similar researches performed in developing countries as Turkey and India, where 36% and 77% of the professionals, respectively, graduated in less than 10 years [16, 17]. However, it may diverge from a descriptive study developed in the United States of America (USA), where most of the professionals (56%) affirmed to have graduated in more than 20 years [18].

The second part of the questionnaire inquired the respondents about technical preferences during the endodontic treatment. The use of rubber dam isolation, that should be mandatory to perform RCT, have been neglected by some professionals (4.5%), or mentioned to be occasionally employed (19.6%). A similar result was found by a study in the USA, where 60% of the general dentists affirmed to always use rubber dam [18]. Interestingly, in the present study, the constant use of rubber dam was significantly more prevalent among postgraduated professionals (75.9%). This fact should be attributed to the higher consciousness acquired by these professionals during their postgraduation programs in order to provide more predictable success rates for the endodontic treatment. On the other hand, developed and developing countries such as England and India, respectively, have demonstrated a low incidence of rubber dam use to perform root canal treatment (30% and 27%, respectively) [13, 17]. Similar alarming data were also observed in Lithuania, where only 12% of the general dentists reported the use of rubber dam isolation during RCT as clinical protocol [19].

Concerning the instrumentation technique, most of the respondents affirmed to use the “crown-down” technique for cleaning and shaping the root canals (83.9%), resembling England and the USA professionals that also reported to adopt this technique (58% and 79.1, respectively) [13, 18]. On the other hand, Iranian and Saudi Arabian dentists mentioned the “Step-back” technique as the most employed in their Endodontics practice for cleaning and shaping root canals (43% and 42%, respectively) [20, 21]. Besides the protocols adopted by the professionals, new instruments have been constantly launched in the market in an attempt to improve the quality and efficiency of root canal treatment [22, 23]. In the present investigation, most of the participants reported to use endodontic hand files routinely (86.9%), mainly due to their indispensable use during the root canals exploration. At the same time, shaping the root canals with rotary/reciprocating NiTi files were mentioned by 58.3% of the respondents, showing that mechanization of RCT still did not replace root canals tactile sensation and negotiation with hand files. Worth mentioning, the use of NiTi instruments in rotary or reciprocating motions for root canal treatment was 71% more prevalent (*p* < 0.05) among the professionals that had attended a postgraduation course in Endodontics, probably due to the fact that CEPEs have been including the learning and practicing of new technology. This data was similar to the findings of a study performed in the USA where the use of NiTi systems were more likely adopted by recent graduated professionals (59.9%) [18]. Amongst the NiTi systems, the reciprocating files were reported in our study as the preferred motion to prepare the root canal (53.3%) followed by the ProTaper rotary system (41.7%). ProTaper files were also described as the first choice by professionals from India, Iranian and Wales (38%, 19%, and 37%, respectively) [14, 17, 20]. Besides the mentioned files, NiTi alloys were recently improved during their fabrication process through thermal surface treatments (*e.g.* R-phase, M-Wire and Controlled Memory NiTi endodontic files), increasing the resistance to cyclic fatigue fracture when compared to conventional (untreated) NiTi files [22, 23]. Despite this progress, only 20% of the respondents reported the use of the thermal treated NiTi files, possibly due to its high cost and/or low percentage of specialists.

In addition to the mechanical preparation of root canals, chemical substances play an important role in the reduction of infection, removal of residual tissues, and also assisting on the instrument's movement. For decades, NaOCl has been largely employed in different countries with widespread preference in the USA and Lithuania (93% and 62.6%, respectively) [18, 19]. Similarly, in the present investigation, most of the professionals described the use of NaOCl (90.4%) for root canal cleaning. A contrary finding was observed in a study designed in India, showing only 33% of the professionals adopting NaOCl as irrigant solution [17]. Similarly, in Saudi Arabia, more than half of the dentists (55%) reported to use saline solution as the first choice for root canals therapy [21] (Table 2).

Intracanal medications have been also used for antimicrobial and conservative pulp therapy purposes, representing different preferences among professionals. In order to achieve all of these requirements, calcium hydroxide has been the most employed intracanal medication in most countries (Lithuania—87%; India—62%; Turkey—53%; Saudi Arabia—85.7%) [16, 17, 19, 21]. Comparably, in the present investigation, 74.3% of the dentists reported to use this material as the first choice for intracanal medication. Conversely, unusual data were reported in a research performed in the USA, where only 19% of the respondents mentioned to use calcium hydroxide as intracanal medicament [18].

Collectively, the present study detected a lack of professionals performing endodontic treatment in the investigated cities of the state of Sergipe. To overcome this problem, a new Dental School was recently introduced in the city of Lagarto (Sergipe) as an extension of the Federal University of Sergipe, since Lagarto represents an inner city located closer to places where it was detected the lack of professionals performing endodontic treatment. Additionally, not only the graduation of more professionals in the region is relevant but also it is expected that these dentists improve their awareness regarding the endodontic treatment as well as the need for updating their knowledge to new technologies available in the Endodontics field, thereby improving the quality and the comfort of the treatment for the patients.

Taking all together, the results of the present study show a tendency in the use of new technologies in Endodontics and also the care with biosafety, including the use of rubber dam to perform the root canal, even in areas involving a low-income population. Furthermore, the data of this study show the concern of most of the professionals to seek knowledge through continuous education programs in order to perform the endodontic treatment following the new available concepts. Meanwhile, a future comparative investigation including more states of Brazil with different sociodemographic conditions may play a more interesting profile of endodontic treatment in the country.

## Figures and Tables

**Table 1:** Sociodemographic and professional qualification of dentists who participated in the study (*n* = 199).

	Frequency (%)
Gender	
Female	59.8
Male	40.2
Age (years)	
20–30	42.2
31–40	31.6
41–50	15.6
>50	10.6
Graduation school	
Public	57.8
Private	42.2
Years of practice	
1–5	38.7
6–10	23.6
11–15	12.1
16–20	7.5
>20	18.1
Endodontics education	
Longer programs (PhD, MSc)	47
Shorter programs	28.5
None	24.5
Qualification in the last 2 years	
Yes	73.9
No	26.1

**Table 2:** Routine endodontic practice by dentists in Bahia and Sergipe, Brazil.

	Frequency (%)
Use of rubber dam	
Always	75.9
Sometimes	19.6
Never	4.5
Type of anesthesic	
Lidocaine	67.8
Articaine	17.1
Mepivacaine	11.6
Lidocaine + articaine	5
Lidocaine + mepivacaine	5
Mepivacaine + articaine	4
Lidocaine + mepivacaine + articaine	2
Others	18.1
Preparation technique	
Crown-down	83.9
Step-back	16.1
Instruments	
Hand endodontic files	86.9
Gates-glidden burs	67.3
Apical locator	62.8
Rotary endodontic files	58.3
Others	32.7
Type of rotary file	
Reciprocating files	53.3
Protaper file	41.7
Easy ProDesign	21.6
MTwo system	5.5
Easy logic	14.58
Easy reciprocating	4.5
Twisted file	1
i-race (FKG)	0.5
Irrigant solution	
NaOCl	90.4
EDTA	84.9
Saline solution	25.1
Chlorhexidine	10
Hydrogen peroxide	5
Others	6
Intracanal medicament	
Ca(OH)_2_	74.3
PMCC	54.3
Otosporin	30.2
Tricresol	17.6
Formocresol	14.6
NaOCl	11.6
Others	16.6
Timing of intracanal medicament	
1–3 days	5
4–7 days	50.3
>7 days	44.2
No answer	0.5

**Table 3:** Crude prevalence ratios (PRs) for factors associated with dentists that performed a Continuous Education Program in Endodontics, from cities of Sergipe and Bahia, Brazil (*n* = 199). Poisson regression analysis (*p* < 0.05).

Independent variable	Continuous education, *n* (%)		PR (IC 95%)	*p*
	No	Yes		
Graduation school				
Public	27 (13.6)	88 (44.2)	1	0.664
Private	22 (11.1)	62 (31.2)	0.97 (0.82–1.14)	

Years since graduation				
>20	7 (3.5)	29 (14.6)	1	0.008
16–20	1 (0.5)	14 (7.0)	1.16 (0.94–1.43)	
11–15	3 (1.5)	21 (10.6)	1.09 (0.87–1.35)	
6–10	9 (4.5)	38 (19.1)	1.00 (0.81–1.24)	
1–5	29 (14.6)	48 (24.1)	0.77 (0.61–0.98)	

Perform RCT using the rotary system				
No	43 (21.6)	53 (26.6)	1	<0.001
Yes	6 (3.0)	97 (48.7)	1.71 (1.42–2.01)	

Endodontic qualification in the last 2 years				
No	15 (7.5)	37 (18.6)	1	0.436
Yes	34 (17.1)	113 (56.8)	1.08 (0.89–1.31)	

Perform RCT using rubber dam isolation				
Never	4 (2.0)	5 (2.5)	1	0.005
Always	26 (13.1)	125 (62.8)	1.49 (0.83–2.69)	
Sometimes	19 (9.5)	20 (10.1)	0.92 (0.48–1.79)	

Perform RCT of necrotic teeth with periapical lesion in one appointment				
No	47 (23.6)	111 (55.8)	1	<0.001
Yes	2 (1.0)	39 (19.6)	1.35 (1.20–1.53)	

Perform RCT of necrotic teeth without periapical lesion in one appointment				
No	40 (20.1)	69 (34.7)	1	<0.001
Yes	9 (4.5)	81 (40.7)	1.42 (1.21–1.67)	

Total time used to perform RCT of incisive teeth in one appointment				
Do not perform	17 (8.5)	8 (4.0)		
≤30 min	9 (4.5)	35 (17.6)	1	0.560
30–60 min	19 (9.5)	87 (43.7)	1.49 (0.72–3.10)	
60–90 min	2 (1.0)	17 (8.5)	1.37 (0.67–2.81)	
>90 min	2 (1.0)	3 (1.5)	1.33 (0.64–2.75)	

Total time used to perform RCT of premolar teeth in one appointment				
Do not perform	21 (10.6)	14 (7.0)		
≤30 min	1 (0.5)	15 (7.5)	1	0.281
30–60 min	14 (7.0)	73 (36.7)	1.10 (0.75–1.62)	
60–90 min	10 (5.0)	40 (20.1)	1.15 (0.79–1.68)	
>90 min	3 (1.5)	8 (4.0)	1.29 (0.88–1.89)	

Total time used to perform RCT of molar teeth in one appointment				
Do not perform	27 (13.6)	18 (9.0)		
≤30 min	0 (0.0)	1 (0.5)	1	<0.001
30–60 min	1 (0.5)	28 (14.1)	1.28 (1.07–1.52)	
60–90 min	5 (2.5)	61 (30.7)	1.33 (1.12–1.59)	
>90 min	16 (8.0)	42 (21.1)	1.38 (1.18–1.62)	

Number of complications by performing RCT in one appointment				
More than three	37 (18.6)	89 (44.7)	1	<0.001
Up to two	10 (5.0)	32 (16.1)	0.93 (0.76–1.14)	
None	2 (1.0)	29 (14.6)	1.23 (1.01–1.49)	

RCT: root canal therapy.

## Data Availability

The data used to support the findings of this study are available from the corresponding author upon request.
